# AC104024.2/miR‐30a‐5p Axis Regulates Malignant Progression of Breast Cancer Through a Depression‐Related Gene, MAP2K3

**DOI:** 10.1155/ancp/6608116

**Published:** 2026-10-22

**Authors:** Jun Li, Jie Sun, Lili Jiang, Xue Bai, Jiyue Gao, Zuowei Zhao

**Affiliations:** ^1^ Department of Breast Surgery The Second Hospital of Dalian Medical University Dalian Liaoning China dlmedu.edu.cn

**Keywords:** AC104024.2/miR-30a-5p axis, breast cancer, ceRNA, depression, MAP2K3

## Abstract

**Background:**

Breast cancer (BC), a malignant tumor, is characterized by high incidence and poor prognosis among women worldwide. Long noncoding RNAs (lncRNAs) and microRNAs (miRNAs) can regulate the initiation and progression of BC through competitive endogenous RNA (ceRNA) mechanisms. Despite depression being an important factor affecting patients with BC, the ceRNA regulatory network involving depression‐related genes in BC remains unclear.

**Methods:**

In this study, we explored the role of the AC104024.2/miR‐30a‐5p axis in BC progression by targeting the depression‐related gene mitogen‐activated protein kinase kinase 3 (MAP2K3). We used depression‐like mouse models to confirm the role of MAP2K3 in modulating depressive behavior. Additionally, gene set enrichment analyses (GSEA) and phenotypic experiments were conducted to verify the effects of the AC104024.2/miR‐30a‐5p/MAP2K3 axis on BC cell proliferation, migration, and apoptosis. Luciferase reporter assays were performed to demonstrate the ceRNA function of AC104024.2 for miR‐30a‐5p.

**Results:**

Our results showed that the AC104024.2/miR‐30a‐5p axis promotes the malignant progression of BC by regulating MAP2K3. In depression‐like mouse models, MAP2K3 was identified as a key gene modulating depressive behavior. The AC104024.2/miR‐30a‐5p/MAP2K3 axis significantly influenced BC cell proliferation, migration, and apoptosis. Luciferase reporter assays confirmed that AC104024.2 acts as a ceRNA for miR‐30a‐5p, thereby affecting MAP2K3 expression. Rescue experiments suggested that the regulatory effects of AC104024.2 on BC malignancy are at least partly dependent on MAP2K3.

**Conclusion:**

This study uncovered the role of the depression‐related ceRNA network in BC progression and identified AC104024.2 and MAP2K3 as potential biomarkers for the diagnosis and treatment of BC. These findings provide significant clinical implications and applications for the management of BC.

## 1. Introduction

Breast cancer (BC) remains a leading cause of morbidity and mortality among malignant tumors in women worldwide [1]. Existing research suggests that BC progression is affected by various factors: on the one hand, biological factors such as genetics and hormone level disorders can significantly increase the risk of BC [2]. On the other hand, a strong association of BC with various lifestyle and environmental risk factors, such as poor dietary habits, tobacco use, alcohol consumption, and physical inactivity, has been demonstrated [3]. Recent advances in multidisciplinary research have enhanced our insights into the molecular pathogenesis of BC; however, challenges related to managing its recurrence and metastasis persist [4]. Given these challenges, it is critical to investigate novel mechanisms of pathogenesis and identify potential therapeutic targets in BC research to provide patients with more targeted and effective personalized therapies.

Notably, the impact of psychological and mental health factors on BC has been increasingly recognized, among which “depression” is regarded as a potential and important factor affecting the progression and treatment effect of BC [5]. For patients with BC, depression weakens their compliance and tolerance to treatment, affects normal endocrine and immune functions, and may accelerate the malignant progression of BC [6]. Clinical research reports have also pointed out that patients with BC suffering from depression often find it difficult to actively cooperate with long‐term treatment, resulting in a decreased reexamination rate and untimely disease monitoring, ultimately increasing the risk of tumor metastasis or recurrence [7]. Paying attention to and intervening in depression is a meaningful entry point for the treatment of BC.

Among the various molecules implicated in BC, mitogen‐activated protein kinase kinase 3 (MAP2K3) has garnered significant attention due to its pivotal role in the mitogen‐activated protein kinase (MAPK) signaling pathway [8]. As a key signaling pathway transmitting external stimuli and regulating gene expression and cellular functions, the MAPK pathway is crucial in processes, including cell growth, differentiation, motility, and apoptosis [9]. However, with continuous investigations into the molecular mechanisms of the malignant progression of BC, researchers have found that MAP2K3 may be important for BC cell invasion and metastasis [10]. MAP2K3 is crucial in the pathogenesis of neuroendocrine diseases [11]. However, systematic reports on the upstream signals MAP2K3 that are activated or inhibited through, and whether they serve a bridging role in integrating depression or stress factors, are lacking. Therefore, we hypothesized that MAP2K3 could serve as a “connector” linking depression‐associated signaling pathways with the oncogenic processes in BC cells, providing potential molecular targets for subsequent prevention and treatment strategies.

With the development of molecular biology and genomics technology, the role of noncoding RNA (ncRNA) has increasingly become prominent [12]. NcRNA includes microRNAs (miRNAs) and long ncRNAs (lncRNAs) [13]. Although they do not directly encode proteins, ncRNAs are important in posttranscriptional regulation [14]. The competitive endogenous RNA (ceRNA) theory holds that various RNA molecules, including mRNA, lncRNA, and circRNA, competitively bind to these miRNAs if they share the same miRNA binding sites, resulting in a dynamic regulatory balance on a macro level [15, 16]. When a certain lncRNA is expressed in large quantities along with key miRNAs, the competing mRNA will reduce the chance of degradation or inhibition by miRNAs, resulting in the upregulation of the expression and function of the mRNA [17]. Recent studies have progressively confirmed the regulatory role of the ceRNA network in various cancers.

Our findings highlight the pivotal role of MAP2K3, identified through a mouse depression model, in regulating both emotional responses and BC malignancy. Specifically, we demonstrate that AC104024.2 functions as a ceRNA by “sponging” miR‐30a‐5p, thereby modulating the MAP2K3 expression. Functional assays confirmed that the AC104024.2/miR‐30a‐5p/MAP2K3 axis influenced BC cell proliferation, migration, and apoptosis. Moreover, mechanistic analyses, including reporter gene and rescue experiments, provided strong evidence for the dependency of AC104024.2’s oncogenic effects on MAP2K3. Overall, our findings highlight the critical role of MAP2K3 in both cancer biology and depression, providing new perspectives on potential therapeutic targets for controlling BC progression.

## 2. Methods

### 2.1. Data Collection

Data from 1231 patients with BC, including clinical information, were obtained from the Cancer Genome Atlas (TCGA) database. The expression profiles of mRNAs, lncRNAs, and miRNAs analyzed in this study were also sourced from the same dataset.

### 2.2. Differential Expression Analysis

In our study, differential expression analysis was performed to identify miRNAs, mRNAs, and lncRNAs exhibiting significant changes in expression. We employed the “limma” package (R, version 3.52.4) to analyze and filter these molecules based on their log fold change (logFC) and associated *p*‐values.

### 2.3. Univariate Cox Analysis

Univariate Cox analysis is employed in survival studies to assess the effect of single variables on patient survival or recurrence rates. Utilizing the Cox proportional hazards model, this method yields risk ratios, significance levels (*p*‐values), and confidence intervals, identifying variables significantly associated with survival outcomes.

### 2.4. Consensus Clustering

Consensus clustering analysis was conducted using the expression data of selected genes using the “ConsensusClusterPlus” R package for clusters, *k* = 2–9, and iterating 1000 times to ensure robust grouping. The consensus matrix and CDF curves were evaluated to select the optimal number of clusters.

### 2.5. Gene Set Enrichment Analysis (GSEA)

To understand the biological relevance of differentially expressed genes, GSEA was performed using the DAVID database. This analysis was designed to identify gene sets correlated with specific biological contexts. We employed version 4.4.4 of the “clusterProfiler” R package for this analysis. The enrichment was considered statistically significant if the *p*‐value was less than 0.05, implying that the gene sets were likely to have biological significance.

### 2.6. Construction of the ceRNA Network

miRcode was used to detect lncRNA–miRNA interactions, followed by using TargetScan, mirTarBase, and miRDB databases to map miRNA–mRNA connections to establish a comprehensive ceRNA network. This process enabled the selection of lncRNAs and miRNAs based on their expressional relevance. Significant interactions among these molecules were curated and integrated into a ceRNA network model. Finally, Cytoscape v3.7.2 was used to graphically visualize the network of the potential regulatory pathways.

### 2.7. Quantitative PCR (qPCR)

After harvesting, the RNA of BC cells was extracted with the RNeasy Mini Kit (Qiagen, 74104, Germany). RNA concentration and purity were determined by NanoDrop analysis. The PrimeScript RT Kit (Takara, RR037Q, China) facilitated reverse transcription to cDNA. SYBR Green Master Mix (Thermo Scientific, A46110, USA) was used for qPCR on a QuantStudio 5 system. The primer sequences are provided in Table S1.

### 2.8. Western Blotting

After washing adherent cells with ice‐cold PBS, total protein was extracted using the ProteoExtract Lysis Buffer. The protein concentration of the resulting lysates was determined. Equal amounts of protein were resolved by SDS‐PAGE, and separated proteins were transferred onto a PVDF membrane. Membranes were blocked with 5% nonfat dry milk and incubated overnight at 4°C with primary antibodies, followed by treatment with biotin‐labeled secondary antibodies. Protein bands were visualized using a colorimetric substrate using a chemiluminescent imaging system. The Erk1/2 antibody used, with the catalog number 11257‐1‐AP, was purchased from Proteintech Group (Wuhan, China) and used at a dilution of 1:3000.

### 2.9. CCK8

Cell viability was assessed using the CCK‐8 assay. Cells (8000 per well) were seeded in 96‐well plates and treated following the specified experimental protocol. After treatment, 100 μL of medium containing 10 μL of CCK‐8 reagent (MCE, HY‐K0301, USA) was added to each well. Following incubation at 37°C for 1 h, absorbance was measured at 450 nm using a CYTATION 5 Imaging Reader (BioTek, USA).

### 2.10. Transwell Migration

BT474 cell migration was evaluated in the absence of Matrigel. Cells were detached, resuspended in serum‐free medium, and plated at a density of 5 × 10^4^ per 100 µL in the upper Transwell chamber. The lower chamber contained 600 µL of complete medium with 20% FBS as a chemoattractant. After the specified incubation time, cells that had not migrated remained on the upper surface of the membrane and were carefully removed using a damp cotton swab. Migrated cells on the lower surface were fixed with 3.7% formaldehyde for 15 min, followed by staining with 0.1% safranin for 15 min. After washing with PBS, the membranes were allowed to air‐dry. The number of migrated cells was quantified by counting cells in at least four random fields under a light microscope at 10× or 20× magnification.

### 2.11. Apoptotic Assay

BT474 cells were gently harvested, washed twice with ice‐cold PBS to remove residual culture medium, and resuspended in the binding buffer of the Annexin V‐FITC/PI Apoptosis Detection Kit (MCE, HY‐K1073, USA). Annexin V‐FITC was used to detect phosphatidylserine externalization on apoptotic cells, while propidium iodide (PI) was utilized to identify cells with compromised membrane integrity. After staining, apoptosis was quantified using a BD FACSCanto II flow cytometer (BD Biosciences), and data analysis was performed with FACSDiva Software to evaluate the apoptotic population based on fluorescence intensity.

### 2.12. Cell Culture

The human mammary duct carcinoma cell line BT474 (RRID: CVCL_VL01) was obtained from ATCC (Manassas, VA, USA). Cells were cultured in RPMI 1640, supplemented with 10% FBS (Thermo Scientific, A5256701, USA) and 1% penicillin‐streptomycin. Cells were incubated at 37°C in an atmosphere with 5% CO_2_. Cells were monitored and subcultured regularly to maintain healthy growth.

### 2.13. Transfection

Lipofectamine 3000 (Invitrogen, L3000001, USA) was used to transfect the siRNAs, antisense oligonucleotides (ASOs), mimics, and inhibitors into cells, following the manufacturer’s instructions. The mimics and inhibitors were purchased from Biomics (Nantong, China). The sequences of siRNA are listed in Table S2.

### 2.14. Luciferase Reporter Gene Assay

We constructed wild‐type (WT) and mutant (Mut) plasmids containing the 3′ UTR of AC104024.2 and seeded BT474 cells into 24‐well plates. After 12 h, cells were cotransfected with the Renilla luciferase control plasmid and a plasmid containing the target 3′ UTR using Lipofectamine 3000 (Invitrogen, L3000001, USA). Twenty‐four hours posttransfection, luciferase activity was assessed using the Dual‐Luciferase Reporter Assay System (Promega, E1910, USA). The ratio of Firefly to Renilla luciferase activities was calculated to evaluate the regulatory effects of AC104024.2 on their respective 3′ UTR sequences.

### 2.15. Mice

All animal‐related procedures were conducted following the guidelines proposed by the ethics committee of the Dalian Medical University and in accordance with the ARRIVE Guidelines to ensure comprehensive and transparent reporting of all aspects of the animal study. A total of 21 healthy female nude mice aged 3–4 weeks and weighing 15–18 g were purchased from Pusheng Technology and acclimatized for 7 days before the experiment. Mice were then randomly allocated into a control group (*n* = 5) and an experimental group (*n* = 16) using a computer‐generated randomization list to ensure unbiased grouping. The nude mice in the control group were fed normally without any treatment. The nude mice in the experimental group were fed in a single cage and treated as follows: (1) food deprivation for 24 h; (2) water deprivation for 24 h; and (3) tails were clamped between the roots of rats for 1 min, and the intensity of tail clamping was based on the first whine of rats. Different stress modes were randomly arranged, each stress was administered at least three times, and the whole process lasted 6 weeks. After 4 weeks of stress stimulation, the mice were inoculated with BC cells and maintained for 2 weeks. All procedures were performed by two independent researchers who were blinded to the group allocation during data collection. Subsequently, 1 × 10^7^/mL BC cell suspension was subcutaneously injected into the axilla of nude mice at random, and the inoculation volume was 0.2 mL. The tumor diameter was measured weekly, and the diameter of the highest point of the tumor‐bearing site and the adjacent site was assessed using vernier calipers. The behavioral aspects of chronic stress depression were tested. Mice were euthanized via an intraperitoneal injection of pentobarbital sodium salt (Sigma–Aldrich, P3761, USA) at a dosage of 100 mg/kg body weight. Subsequently, tumors were harvested for protein analysis.

### 2.16. Forced Swim Test (FST)

Mice underwent a preexperimental swim training 24 h before the formal trial. Each mouse was placed into a transparent cylindrical glass container (height ~30 cm; diameter 20 cm) filled with water maintained at (25 ± 5)°C to a depth of 15 cm, preventing the animal from standing on the bottom or escaping. During the formal test, a video camera recorded the 6‐min session, and immobility time (floating with minimal hindlimb paddling to keep the head above water) was scored during the final 4 min using Smart 3.0 software.

### 2.17. Tail Suspension Test (TST)

A piece of adhesive tape was wrapped around the mouse tail, ~1 cm from the tip. The free end of the tape was then attached to a suspension bar so that the tail hung vertically, with the mouse’s head positioned roughly 15 cm above the floor. To prevent tail climbing, which can interfere with immobility scoring, a small plastic tube was placed around the tail near the tape attachment. Each mouse was tested for a single 6‐min session, and immobility time was measured and analyzed during the final 4 min.

### 2.18. Elevated Plus Maze (EPM) Test

The EPM apparatus consisted of two opposing open arms (50 × 10 × 0.5 cm), two opposing closed arms (50 × 10 × 20 cm), and a central open platform (10 × 10 cm). Each mouse was placed onto the central platform facing one of the open arms and allowed to explore the maze freely for 5 min. The primary outcome recorded was the distance traveled within the closed arms for each mouse.

### 2.19. Enzyme‐Linked Immunosorbent Assay (ELISA)

Twenty‐four hours after behavioral testing, mice were fasted and euthanized by cervical dislocation. Blood was collected via orbital enucleation, followed by decapitation to harvest the brain. On ice, ~0.2 g of brain tissue was homogenized in cold saline and centrifuged to obtain the supernatant. The concentration of the neurotransmitter 5‐hydroxytryptamine (5‐HT) in the mice brain was quantified by ELISA following the manufacturer’s instructions for the enzyme‐linked immunoassay kit.

### 2.20. CRISPR/Cas9 Vector Design and Cell Transfection for lncRNA AC104024.2

Specific gRNAs targeting lncRNA AC104024.2 were designed using the Chop–Chop software (https://chopchop.cbu.uib.no). The Cas9 expression vector pSPCs9 (containing plasmid PX459 Turbo GFP) was employed, and oligonucleotides for two distinct gRNAs were synthesized to cleave AC104024.2 at predefined sites. The resulting PX459‐AC104024.2‐gRNA1 and PX459‐AC104024.2‐gRNA2 plasmids were transfected into BT474 cells using the CRISPRMAX transfection reagent in Opti‐MEM medium, following the manufacturer’s protocol. After 6 h, the medium was replaced with complete RPMI 1640, and cells were cultured for 48 h. GFP expression was monitored under fluorescence microscopy at both 24 and 48 h posttransfection. Transfection efficiency was validated through GFP fluorescence detection and T7 endonuclease I (T7EI) assay. Cells exhibiting positive GFP expression were selected for downstream experiments. The knockout efficiency of AC104024.2 was subsequently confirmed by qPCR analysis.

### 2.21. RNA Immunoprecipitation qPCR (RIP‐qPCR)

Gene expression profiles were quantified using the Dynabeads mRNA Purification Kit (Catalog Number 61,006; Invitrogen). Total RNA was first isolated for analysis. Immunoprecipitation was performed by incubating the RNA with either anti‐AGO2 (Catalog Number ab156870; Abcam) antibodies or a nonspecific control IgG (Catalog Number ab172730; Abcam), under rotation at 4°C for 2 h. Pierce Protein A/G Magnetic Beads (Catalog Number 88,803; Thermo Scientific) were then added to capture the RNA‐antibody complexes. The beads were collected using a magnetic separator, washed three times in 1× immunoprecipitation buffer supplemented with RNase inhibitor, and treated with proteinase K to elute the RNA. Following digestion, 5 μg of glycogen and 2.5 volumes of 100% ethanol in 3 M sodium acetate were added to facilitate precipitation at −80°C overnight. Precipitated RNA was recovered and subjected to qPCR for mRNA quantification.

### 2.22. Statistical Analysis

Statistical analyses were performed using SPSS version 22.0 (SPSS Inc., Chicago, IL, USA) and GraphPad Prism version 8.0 (GraphPad Software, La Jolla, CA, USA). For comparisons between two groups, a *t*‐test was used to assess mean differences. For multiple‐group comparisons, one‐way ANOVA was used, and Pearson’s correlation coefficient was used to assess the linear relationship between continuous variables. A receiver operating characteristic (ROC) curve analysis was employed to assess the area under the curve (AUC) to determine the sensitivity and specificity of the biomarkers to evaluate the potential of specific biomarkers or variables for clinical prediction. Kaplan–Meier survival curves were constructed to estimate survival probability across different groups, and the significance of survival differences was assessed using the log‐rank test. A *p*‐value less than 0.05 was considered statistically significant for all tests.

## 3. Results

### 3.1. Depressive Behavior Downregulates MAP2K3 Expression and Affects the Progression of BC In Vivo

Depression may be involved in BC progression and treatment efficacy. To investigate how depression may influence BC progression, we constructed a depression model of nude mice using chronic unpredictable mild stress (CUMS). The results of behavioral tests prior to tumor cell inoculation confirmed the successful construction of the depression model (Figure 1A). Subsequently, data analysis revealed a gradual body weight loss in the experimental group both before and after tumor cell inoculation (Figure 1B). Behavioral tests conducted after tumor cell inoculation further demonstrated that mice maintained persistent depressive states (Figure 1C). Compared to the control group, the depression mouse model exhibited a significant increase in tumor weight after tumor cell inoculation (Figure 1D). Additionally, the content of 5‐HT in the brain of the mice was analyzed postinoculation, which also supported the persistence of depressive states (Figure 1E). However, the relative levels of cortisol further demonstrated that depressive behavior induced a concurrent systemic stress response (Figure S1A). It has been documented that the expression of Erk1/2 is indicative of depressive states in mice. Therefore, we also examined the expression of Erk1/2 and found that, compared to the control group, it was indeed upregulated in the depressed mice (Figure 1F). MAP2K3 can regulate the invasion and metastasis of BC cells; therefore, we choose MAP2K3 for further investigation. Western blot analysis of tumor samples from mice revealed elevated MAP2K3 protein expression compared with the controls (Figure 1G), suggesting that depressive behavior upregulated MAP2K3 expression in BC. We performed a Kaplan–Meier survival analysis comparing the high‐ and low‐MAP2K3 expression groups to assist in evaluating the clinical prognostic significance of MAP2K3 in BC. The results showed that patients with high MAP2K3 expression had poorer survival (Figure 1H). In addition, we divided the expression data of BC in the TCGA database into high‐ and low‐MAP2K3 expression groups to perform differential gene expression analysis. We obtained 756 upregulated genes and 545 downregulated genes (Figure 1I,J). GSEA identified significant enrichment of differentially expressed genes in the proliferation, migration, and apoptosis pathways (Figure 1K). We inferred that depressive behavior regulated the malignant progression of BC by MAP2K3.

Figure 1Depressive behavior regulates breast cancer progression. (A) Behavioral tests of depressive‐ and anxiety‐like behaviors in mice before breast cancer cell inoculation. Immobility time in the forced swim test (FST) and tail suspension test (TST), and closed‐arm time in the elevated plus maze (EPM). (B) Weight changes before and after breast cancer cell inoculation on control and stress group mice. (C) Behavioral tests of depressive‐ and anxiety‐like behaviors in mice after breast cancer cell inoculation. Immobility time in the forced swim test (FST) and tail suspension test (TST), and closed‐arm time in the elevated plus maze (EPM). (D) Tumor burden after breast cancer cell inoculation on control and stress group mice. (E) The content of 5‐HT in the brain of the mice. (F) Detecting Erk1/2 expression levels in breast cancer tumors of control and stress group mice. (G) Detecting MAP2K3 expression levels in breast cancer tumors of control and stress group mice. (H) Kaplan–Meier survival analysis of overall survival (OS) in breast cancer patients stratified by MAP2K3 expression levels. (I) Volcano plot shows differential expressed gene analysis results between high and low MAP2K3 expression groups in TCGA‐BRCA. (J) Heat map shows differential expressed genes between high and low MAP2K3 expression groups in TCGA‐BRCA. (K) Bubble plot shows enrichment pathway of differential expressed genes between high and low MAP2K3 expression groups. TST, tail suspension test; EPM, elevated plus maze test; FST, forced swim test. ^∗^, *p* < 0.05,  ^∗∗∗^, *p* < 0.001.

**Figure:**
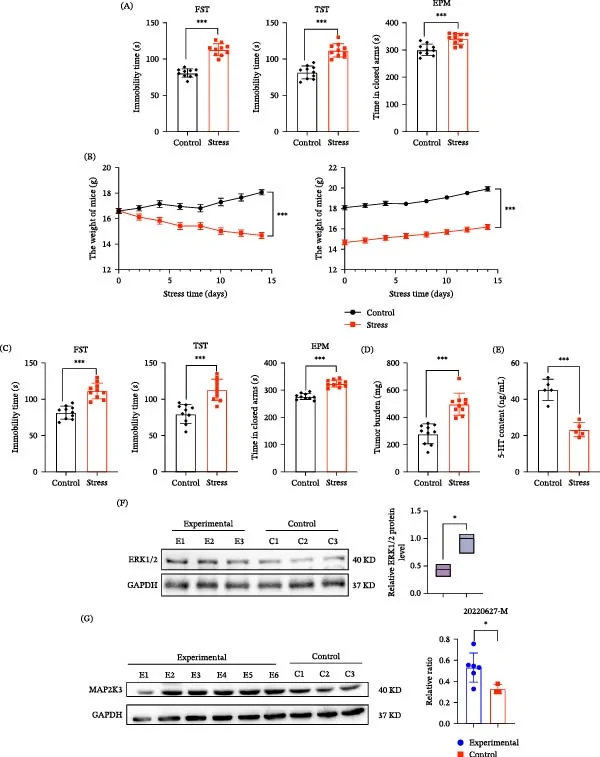

**Figure:**
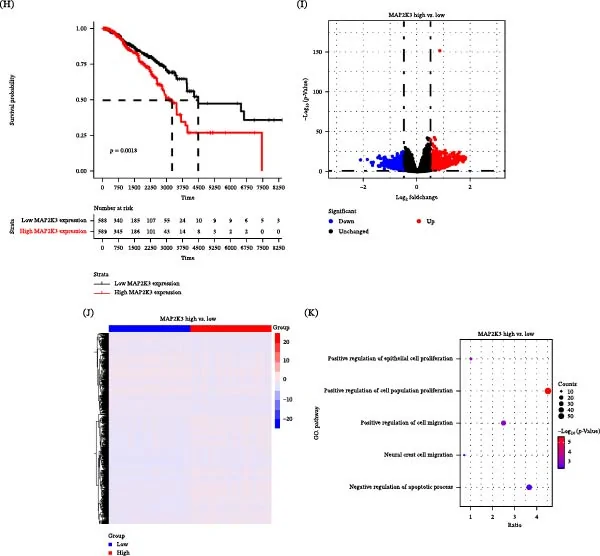

### 3.2. MAP2K3 Promotes Proliferation and Migration and Inhibits Apoptosis in BC Cells

To further clarify the regulatory role of MAP2K3 in BC, we used the corresponding plasmids to overexpress MAP2K3. CCK8, Transwell, and apoptosis assays were performed to assess cell proliferation, migration, and apoptosis to investigate the effects of MAP2K3 on BC progression. Overexpression of MAP2K3 significantly increased BC cell proliferation and migration and inhibited apoptosis compared with the control (Figure 2A–C). These findings suggest that MAP2K3 plays a key role in promoting cell proliferation and migration and reducing apoptosis in BC cells.

**Figure 2:**
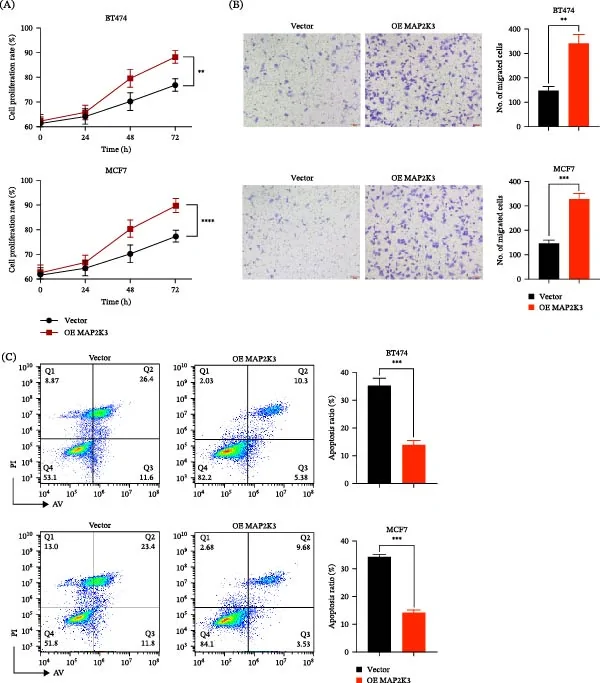
MAP2K3 can regulate proliferation, migration, and apoptosis of breast cancer cells. (A) CCK8 assay shows the proliferation ability of BT474 cells after overexpressing MAP2K3. (B) Transwell assay shows the invasion ability of BT474 cells after overexpressing MAP2K3. (C) Apoptosis assay shows the apoptosis of BT474 cells after overexpressing MAP2K3. The statistical method is *T*‐test. Values were expressed as the mean ± SD from three experiments, and the asterisk indicates the statistical significance compared to the controls ( ^∗∗^ *p* < 0.01, ^∗∗∗^ *p* < 0.001, ^∗∗∗∗^ *p* < 0.0001).

### 3.3. Screening lncRNAs and miRNAs Related to the Progression of BC

Tumor biomarkers are crucial for the early diagnosis of cancer, with their expression levels often undergoing significant alterations during tumor progression. Many of these biomarkers function as oncogenes or tumor suppressor genes and regulate the progression of tumors. However, the role of ceRNA networks involving these genes in BC progression remains poorly understood. We analyzed the differential expression of lncRNAs and miRNAs in 130 paired BC and adjacent normal tissues to investigate the underlying mechanisms. The analysis identified 984 upregulated and 1455 downregulated lncRNAs, along with 67 upregulated and 73 downregulated miRNAs (Figure 3A,B). Next, we performed univariate Cox analysis on 1231 BC samples. We identified 586 oncogenes and 443 tumor suppressor genes among lncRNAs and 57 oncogenes and 61 tumor suppressor genes among miRNAs (Figure 3C).

**Figure 3:**
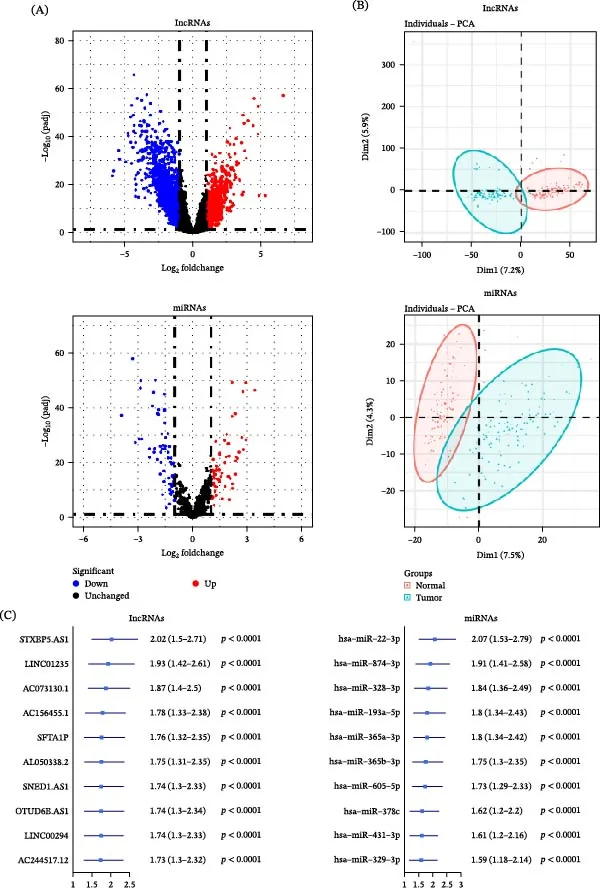
The results of univariate cox and differential expression analysis of lncRNAs and miRNAs from the TCGA database. (A) Volcano plots of differential expression analysis of lncRNAs and miRNAs. (B) PCA depicted the distribution for primary solid tumor and solid tissue normal of lncRNAs and miRNAs. (C) The top 10 results of univariate cox in lncRNAs and miRNAs.

### 3.4. AC104024.2 and miR‐30a‐5p Are Key Genes Regulating MAP2K3 In Vitro

Through in vivo experiments, we found that MAP2K3 is a crucial gene for depression to regulate BC. Based on the results of univariate Cox analysis and the MAP2K3 gene, we constructed a ceRNA network to investigate the regulatory mechanisms underlying the malignant progression of BC. This network comprised six lncRNAs, one miRNA, and six distinct interaction axes (Figures 4A and S2A). AC009812.4/miR‐30a‐5p, AC012085.2/miR‐30a‐5p, AC104024.2/miR‐30a‐5p, AP000265.1/miR‐30a‐5p, AP000766.1/miR‐30a‐5p, and SCAT8/miR‐30a‐5p axes may all regulate MAP2K3 (Figure 4B). The above six lncRNAs were screened to explore the main lncRNA regulating MAP2K3. We performed correlation and survival analyses to investigate the roles of AC009812.4, AC012085.2, AC104024.2, AP000265.1, AP000766.1, and SCAT8. The results of correlation analysis with MAP2K3 showed that AC104024.2 and SCAT8 were positively and strongly correlated with MAP2K3 (Figure 5A). Survival analysis revealed that elevated expression levels of AC104024.2 and SCAT8 were significantly correlated with poorer prognosis (Figure 5B). The AUC values for AC104024.2 and SCAT8 were 0.564 and 0.551, respectively (Figure 5C). To identify key long ncRNAs (lncRNAs),ASOs were employed to specifically downregulate AC104024.2 and SCAT8. Subsequently, we performed CRISPR‐based knockout of six lncRNAs. The results demonstrated that knockout of AC104024.2 significantly reduced the expression level of MAP2K3, whereas no significant changes were observed upon knockout of the other lncRNAs (Figure 5D). These findings suggest that AC104024.2 plays a central role in regulating the MAP2K3 expression. Thus, the AC104024.2/miR‐30a‐5p/MAP2K3 axis may be a crucial ceRNA network involved in the pathogenesis of BC, potentially linked to its depressive phenotype.

**Figure 4:**
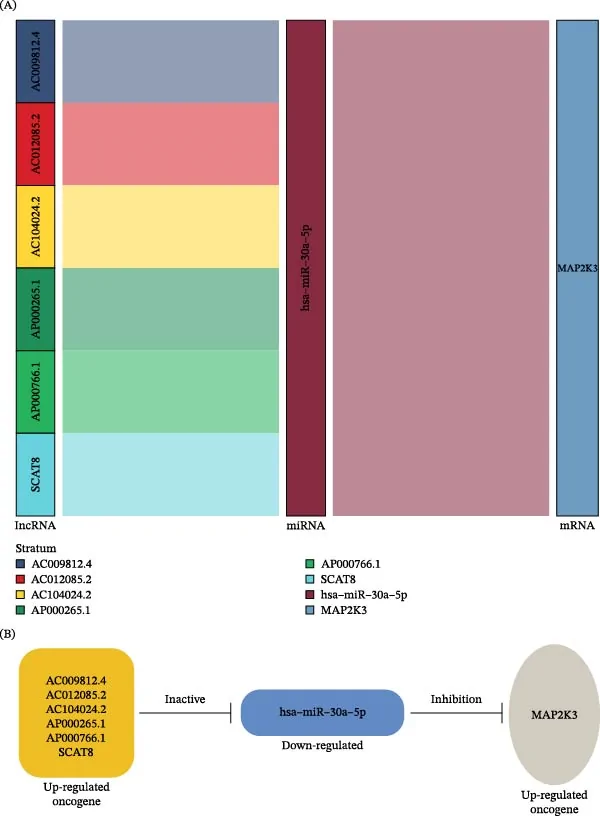
Construction of breast cancer–related ceRNA network by integrated analysis. (A) lncRNA‐miRNA‐mRNA regulatory axes extracted from this ceRNA network. (B) The lncRNA‐miRNA‐mRNA pattern diagram.

Figure 5AC104024.2/miR‐30a‐5p/MAP2K3 may be an important ceRNA axis for depression to regulate breast cancer. (A) Pearson correlation coefficient analysis between MAP2K3 and six potential lncRNAs. (B) Survival analysis in the high and low expression lncRNA groups. (C) The ROC curves of the potential lncRNAs. (D) RT‐qPCR assays were used to detect the expressions of MAP2K3 mRNA levels in BC cells with potential lncRNA knockout by CRISPR. The statistical method is *T*‐test. Values were expressed as the mean ± SD from three experiments, and the asterisk indicates the statistical significance compared to the controls (NS, not significant,  ^∗^ *p* < 0.05,  ^∗∗∗∗^ *p* < 0.0001).

**Figure:**
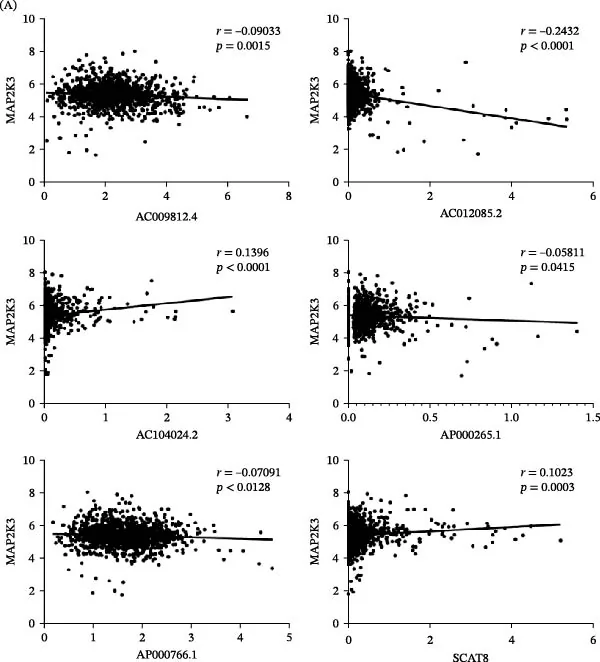

**Figure:**
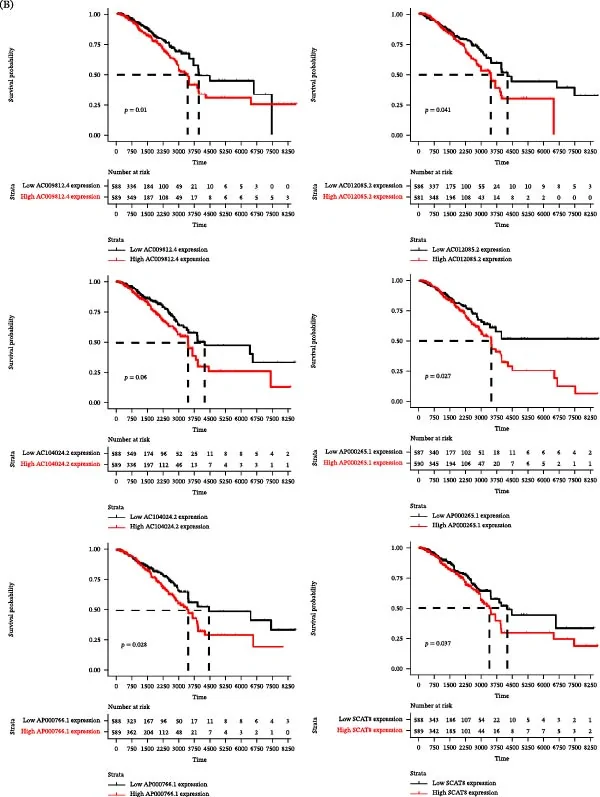

**Figure:**
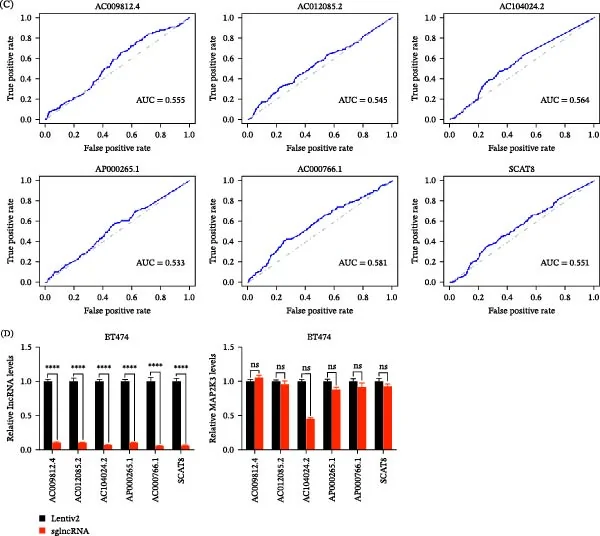

### 3.5. The ceRNA Axis AC104024.2/miR‐30a‐5p/MAP2K3 Regulates Malignant Progression of BC

Building on predictive analysis, the AC104024.2/miR‐30a‐5p/MAP2K3 axis has been proposed as a potential regulator of BC initiation and progression. However, the precise role of this axis in the malignant transformation of BC remains unclear. BC samples were stratified based on AC104024.2 expression levels into high and low expression groups and subjected to differential expression analysis and GSEA. Similarly, miR‐30a‐5p expression was categorized into high and low subgroups. The analysis revealed that genes associated with AC104024.2 and miR‐30a‐5p were predominantly involved in pathways related to apoptosis and cell proliferation (Figures 6A and S3). To further verify the regulatory effects of AC104024.2 and miR‐30a‐5p on apoptosis and proliferation of BC, CCK8 assays and apoptosis experiments were conducted after inhibiting AC104024.2 by ASO and overexpressing miR‐30a‐5p using a mimic. When AC104024.2 was inhibited, the proliferation of BC cells was also inhibited, and the apoptosis of BC cells was increased. Similarly, when miR‐30a‐5p was overexpressed, it inhibited the proliferation and enhanced the apoptosis of BC cells. The findings also align with the results of GSEA (Figure 6B,C). The above results indicate that the AC104024.2/miR‐30a‐5p/MAP2K3 ceRNA axis can regulate the malignant progression of BC.

**Figure 6:**
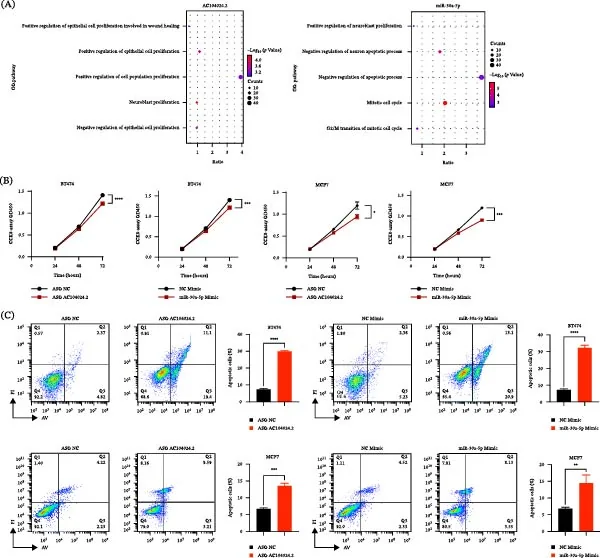
The ceRNA axis AC104024.2/miR‐30a‐5p/MAP2K3 regulates the malignant progression of breast cancer. (A) The bubble diagrams of GO enrichment analysis in AC104024.2 and miR‐30a‐5p by DAVID database. (B) The CCK8 assays of breast cancer cells were performed when AC104024.2 was knocked down by ASO, and miR‐30a‐5p was overexpressed by mimic for 48 h. (C) The cell apoptosis assays of breast cancer cells were performed when AC104024.2 was knocked down by ASO, and miR‐30a‐5p was overexpressed by mimic for 48 h. The statistical method is *T*‐test. Values were expressed as the means ± SD from three experiments, and the asterisk indicates the statistical significance compared to the controls ( ^∗^ *p* < 0.05, ^∗∗^ *p* < 0.01, ^∗∗∗^ *p* < 0.001,  ^∗∗∗∗^ *p* < 0.0001).

### 3.6. AC104024.2 and miR‐30a‐5p Can Regulate MAP2K3 in BC

To further investigate the relationship of AC104024.2, miR‐30a‐5p, and MAP2K3, after knocking down AC104024.2 using ASO, we detected the changes in miR‐30a‐5p and MAP2K3 levels. Knocking down AC104024.2 resulted in the downregulation of MAP2K3 and upregulation of miR‐30a‐5p (Figure 7A) expression. Subsequently, miR‐30a‐5p was overexpressed and inhibited using its corresponding mimic and inhibitor. The expression levels of AC104024.2 and MAP2K3 were assessed, revealing that both were negatively regulated by miR‐30a‐5p (Figure 7B,C). These findings suggest that AC104024.2 and miR‐30a‐5p collaboratively modulate MAP2K3 expression in BC, consistent with the ceRNA regulatory network.

**Figure 7:**
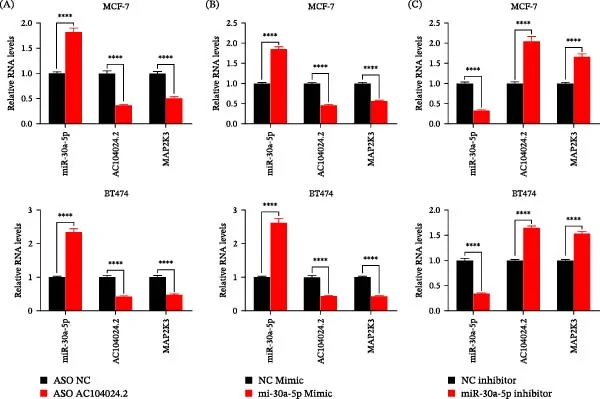
AC104024.2 and miR‐30a‐5p can regulate MAP2K3 in breast cancer. (A) RT‐qPCR assays were used to detect the expression of AC104024.2, miR‐30a‐5p, and MAP2K3 in breast cancer cells treated with AC104024.2 ASO. (B) RT‐qPCR assays were used to detect the expression of AC104024.2, miR‐30a‐5p, and MAP2K3 in breast cancer cells treated with miR‐30a‐5p mimic for 48 h. (C) RT‐qPCR assays were used to detect the expression of AC104024.2, miR‐30a‐5p, and MAP2K3 in breast cancer cells treated with miR‐30a‐5p inhibitor for 48 h. The statistical method is *T*‐test. Values were expressed as the means ± SD from three experiments, and the asterisk indicates the statistical significance compared to the controls ( ^∗∗∗∗^ *p* < 0.0001).

### 3.7. AC104024.2, as the ceRNA of miR‐30a‐5p, Regulates MAP2K3 to Affect the Malignant Progression of BC

To investigate whether AC104024.2 acts as a ceRNA for miR‐30a‐5p, its predicted binding sites for miR‐30a‐5p were mutated, and luciferase assays were conducted to evaluate changes in luciferase activity before and after mutation. miR‐30a‐5p significantly suppressed luciferase activity in WT AC104024.2, with no change in the Mut (Figure 8A,B). To validate the sponge mechanism of the ceRNA, we performed an AGO2 RIP‐qPCR assay. The results showed that, compared with the control IgG antibody, the AGO2 antibody significantly enriched greater amounts of both miR‐30a‐5p and AC104024.2 (Figure 8C). This indicates that miR‐30a‐5p and AC104024.2 form a molecular sponge as ceRNAs. The conclusion that AC104024.2 acts as a ceRNA for miR‐30a‐5p to regulate MAP2K3 was further validated at the molecular rigor through qPCR experiments. In both the control group and the experimental group overexpressing miR‐30a‐5p using mimics, we overexpressed either the WT or the mutated AC104024.2 and examined changes in MAP2K3 expression. In the NC mimic group, overexpressing WT AC104024.2 upregulated MAP2K3 expression, while overexpressing the mutated AC104024.2 failed to upregulate MAP2K3. In the miR‐30a‐5p‐mimic group, neither the WT nor the mutated AC104024.2 had any effect. These results provide further evidence supporting the conclusion that AC104024.2 acts as a ceRNA for miR‐30a‐5p to regulate MAP2K3 (Figure 8D). Next, we performed GSEA to identify genes coregulated by AC104024.2 and MAP2K3 involved in the malignant progression of BC (Figure 8E). These genes were significantly enriched in pathways related to cell proliferation (Figure 8F). To confirm this observation, AC104024.2 was knocked down using ASO in the control and siMAP2K3 groups, and cell proliferation was assessed. AC104024.2 knockdown reduced BC cell proliferation in the control group. This effect could not be observed in the MAP2K3 knockdown group (Figure 8G). Furthermore, we reinforced this conclusion by knocking down AC104024.2 in both the NC‐inhibitor group and the miR‐30a‐5p inhibitor group and examining its impact on proliferation via CCK8 assays. We found that knockdown of AC104024.2 by ASO inhibited BC cell proliferation in the NC‐inhibitor group, whereas it had no effect in the group where miR‐30a‐5p was suppressed, which would strengthen our claim (Figure 8H). These findings suggest that AC104024.2, as a ceRNA of miR‐30a‐5p, modulates the MAP2K3 expression, thereby influencing the malignant progression of BC.

Figure 8As the ceRNA of miR‐30a‐5p, AC104024.2 regulates MAP2K3 and affects the malignant progression of breast cancer. (A) Predicted binding site between miR‐30a‐5p and AC104024.2. (B) Luciferase assays were performed to test the effect of miR‐30a‐5p on wild‐type or mutant AC104024.2 after treating with miR‐30a‐5p mimic for 48 h. (C) The AGO2 RIP‐qPCR assays were used to validate the sponge mechanism of the ceRNA. (D) qPCR analysis of MAP2K3 expression after overexpression of wild‐type (WT) or mutant (Mut) AC104024.2 in cells transfected with either NC mimic or miR‐30a‐5p mimic. (E) Venn diagrams represent the overlapped genes between AC104024.2 and MAP2K3. (F) The bubble diagram of GO enrichment analysis of AC104024.2 and MAP2K3 overlapped genes by DAVID database. (G) The CCK8 results of siNC or siMAP2K3 breast cancer cell lines transfected with AC104024.2 ASO. (H) CCK8 assays were used to test the proliferation effect of knocking down AC104024.2 in both the control group and the miR‐30a‐5p inhibitor group. The statistical methods are *T*‐test and two‐way ANOVA. Values were expressed as the means ± SD from three experiments, and the asterisk indicates the statistical significance compared to the controls (NS, not significant,  ^∗∗∗∗^ *p* < 0.0001).

**Figure:**
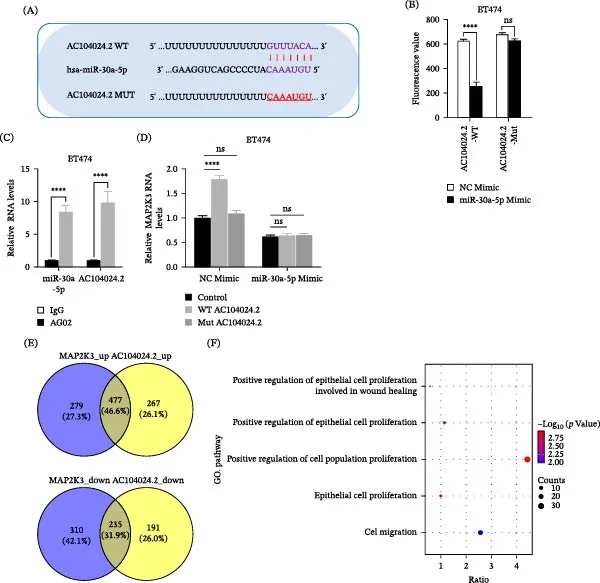

**Figure:**
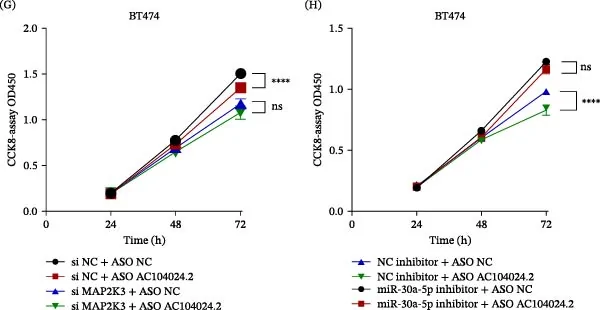

## 4. Discussion

Pathophysiological mechanisms underlying depression, a complex psychiatric and psychological disorder, include imbalances in neurotransmitters and dysregulation of the hypothalamic‐pituitary‐adrenal axis, among other physiological pathways [18]. It can be influenced by social environment and lifestyle factors. In contrast to anxiety‐ or stress‐related disorders, depression often presents as persistent low mood, loss of interest, and reduced motivation, which may trigger symptoms, including sleep disturbances, poor appetite, and cognitive impairment [19]. Clinical data indicate that women facing serious illnesses are more prone to developing depressive symptoms [20]; in particular, individuals diagnosed with BC frequently experience heavier psychological burdens, leading to decreased treatment adherence and slower recovery [21]. In some patients, the depressive state is accompanied by compromised immune responses and elevated inflammatory mediators, creating conditions favorable for tumor cell growth and metastasis [22]. Prolonged depression can exacerbate the tumor microenvironment and promote resistance to chemotherapy and endocrine therapy [23]. Investigating the molecular links between depression and BC may, therefore, offer new clinical approaches for integrated intervention, aiming to improve both physical and psychological outcomes in these patients. Consequently, depression may serve as an essential entry point for more effective BC treatment and management.

Advances in understanding posttranscriptional gene regulation have brought forth the ceRNA network. mRNAs, lncRNAs, and circRNAs can share similar miRNA binding sites, allowing them to compete for miRNA binding, thereby influencing each other’s translation or degradation [24]. This competition creates a dynamic equilibrium at the system level. In this process, lncRNAs frequently act as modulators or sponges, sequestering certain miRNAs and modulating the expression of critical target genes [25]. The importance of ceRNA networks in BC has partially been established, with studies indicating their role in regulating cell cycle progression, metastasis, drug resistance, and immune evasion. This is exemplified by the role of the LINC00466‐hsa‐miR‐204‐NTRK2 axis in invasion [26, 27]. CeRNA networks significantly impact treatment responses; for instance, the LINC00589 network influences trastuzumab treatment outcomes in HER2‐positive BC, and the FTH1P3 pseudogene is linked to paclitaxel resistance [28]. Additionally, these networks are associated with immune cell infiltration in the tumor microenvironment, potentially affecting immunotherapy responses [29]. Complex interactions within ceRNA networks provide a comprehensive view of BC biology, offering new avenues for research, prognostic marker identification, and the development of novel therapeutic strategies.

In this study, we used a mouse model of depression and found that MAP2K3 not only was pivotal to emotional regulation but also significantly impacted the malignant progression of BC. MAP2K3 is a critical kinase within the MAPK signaling pathway, closely associated with cellular stress responses, inflammation, and various survival pathways. Aberrant MAP2K3 expression may disrupt the balance between cell proliferation and apoptosis, thereby affecting tumor development and metastasis [30]. Our findings, integrating the depression model, reveal that MAP2K3 may respond to emotional stress signals and transmit them to intracellular transcription networks, providing a potential molecular route for the interaction between emotional states and tumor progression. MAP2K3 has been implicated in various ceRNA networks and plays a role in different biological processes and diseases. In endometriosis and triple‐negative BC, MAP2K3 has been identified as part of ceRNA networks involving lncRNAs and circRNAs that potentially regulate expression by competing for shared miRNAs. MAP2K3 is involved in immune‐related ceRNA networks, as evidenced by its upregulation in monocyte activation [31–33]. MAP2K3’s role in the MAPK signaling pathway suggests potential ceRNA mechanisms affecting gene expression in this crucial cellular cascade. While these findings highlight the diverse involvement of MAP2K3 in ceRNA networks, many of these inferences are based on bioinformatics analyses and predictive models, necessitating experimental validation to fully understand MAP2K3’s role in ceRNA networks and its implications in different biological contexts. Targeting or blocking MAP2K3‐related signaling in the BC tissue may slow tumor spread and improve patients’ psychological well‐being. MAP2K3 holds promising applications in both BC prognosis and the management of depressive symptoms, laying a foundation for more in‐depth research and clinical interventions. An intriguing question arises when depression, a complex emotional factor, is introduced: The ceRNA network may serve as a molecular bridge by which emotional signals reach tumor cells. In our study, analyses of a depression‐related BC model led to the identification of numerous differentially expressed lncRNAs and miRNAs, and we subsequently focused on the MAP2K3 axis. The competitive interaction between AC104024.2 and miR‐30a‐5p strongly affected BC cell proliferation, migration, and apoptosis. This offers new insights into the interplay between depression and tumor cells.

The present study has some methodological limitations. The UCMS depression model, while representing the current gold standard for preclinical depression research, exhibits variable reproducibility and susceptibility to uncontrolled environmental and genetic factors. Regarding bioinformatics analyses, ceRNA network inference is constrained by the limitations. First of all, algorithmic constraints of sequence‐based miRNA target prediction tools that overlook structural accessibility, subcellular colocalization, and protein‐mediated binding determinants. Besides threshold‐dependent network topology, arbitrary cutoffs for correlation and prediction scores may bias hub node identification and regulatory hierarchy interpretation. These considerations underscore the necessity of experimental validation and integration of emerging technologies to substantiate the regulatory interactions proposed herein and advance our understanding of stress‐responsive molecular networks in depression. Moreover, although our luciferase reporter assay demonstrated that miR‐30a‐5p can directly bind to the predicted site within MAP2K3 mRNA, this approach primarily reflects the possibility of base pairing and does not fully establish the endogenous interaction between miR‐30a‐5p and MAP2K3 mRNA under physiological conditions. Therefore, the endogenous binding between miR‐30a‐5p and MAP2K3 mRNA remains to be fully verified. Future studies could employ RNA pull‐down assays or dual‐luciferase reporter assays with binding‐site mutations to further confirm the direct interaction between miR‐30a‐5p and MAP2K3.

In conclusion, in this study, we established a preliminary framework integrating “emotion‐regulating genes, ceRNA networks, and tumor growth” through molecular and cellular experiments. We elucidated the “depression‐breast cancer” signaling network, which is linked by MAP2K3. We initially confirmed that AC104024.2, as a ceRNA for miR‐30a‐5p, significantly affected the expression of the key gene, MAP2K3, emphasizing the potential role of the AC104024.2/miR‐30a‐5p ceRNA regulatory axis in BC malignant phenotypes. The mouse depression model indicates the importance of MAP2K3 in emotional regulation, while enrichment analysis and knockdown experiments demonstrate that the AC104024.2/miR‐30a‐5p/MAP2K3 axis can regulate BC cell proliferation, migration, and apoptosis. Mechanistically, reporter gene experiments showed that AC104024.2 upregulated MAP2K3 by “sponging” miR‐30a‐5p. More importantly, subsequent “rescue” experiments confirmed that AC104024.2’s effect on BC malignant progression largely depended on MAP2K3, making this pathway a new potential option for clinical intervention. Our research reveals the deep ceRNA network underlying depression in BC and provides new insights for subsequent precision treatment and comprehensive management of patients with BC. With an in‐depth understanding of depression mechanisms and ncRNA regulation, opportunities to develop multitarget comprehensive treatment strategies by targeting this pathway in the future can provide effective diagnostic and treatment options for BC.

## Author Contributions

All authors contributed to the study conception and design. Jun Li, Jie Sun, Lili Jiang, and Xue Bai performed the experiments. Jiyue Gao and Zuowei Zhao performed the data analysis. The first draft of the manuscript was written by Jiyue Gao and Zuowei Zhao.

## Funding

This research was supported by the National Natural Science Foundation of China (Grants 82072934 and 82274296 to Zuowei Zhao).

## Disclosure

All authors commented on previous versions of the manuscript. All authors read and approved the final manuscript. No AI software was used in the preparation of this manuscript, either as a whole or in part.

## Ethics Statement

All animal experiments conformed to the National Health Guide for the Care and Use of Laboratory Animals and were approved by the Animal Experiments Committee of Dalian Medical University (Approval Number AEE24306).

## Conflicts of Interest

The authors declare no conflicts of interest.

## Supporting Information

Additional supporting information can be found online in the Supporting Information section.

## Supporting information


**Supporting Information** The primer sequences were in Table S1. The sequence of siRNAs was in Table S2. The supporting information included supporting figures and supporting figure legends.

## Data Availability

The data generated in this study are available within the article and its supporting data files.
