# Supplementary material for: AC104024.2/miR‐30a‐5p Axis Regulates Malignant Progression of Breast Cancer Through a Depression‐Related Gene, MAP2K3
Source: Anal Cell Pathol (Amst). 2026 Oct 22;2026:6608116. doi: 10.1155/ancp/6608116 (PMC13691419; doi:10.1155/ancp/6608116)
Supplement: Supplementary file 1 [file ANCP-2026-6608116-s001.zip › Supplemental materials.docx]

**FigureS1**

**FigureS2**

**FigureS3**

**Supplementary figure legends**

**FigureS1**

The relative levels of cortisol in the plasma of mice exposed to CUMS and control mice were measured by ELISAs.

**FigureS2**

Construction of ceRNA network by integrated analysis.

**FigureS3**

A. The heatmap and the volcano plot of differential expression analysis of AC104024.2 in low expression group and high expression group.
B. The heatmap and the volcano plot of differential expression analysis of miR-30a-5p in low expression group and high expression group.
